
JOURNAL INFORMATION
==============================
NLM Title Abbreviation: Atlanta Med Surg J (1884)
NLM Journal ID: 101251623

Atlanta Medical and Surgical Journal (1884)

Publisher: Biomedical Journal Digitization Project

ARTICLE INFORMATION
==============================
PMCID: PMC9201845
PMID: 35829602
Article version: 1
Subjects: Original Communications

The Technics of the Operative Treatment of Intestinal Obstruction

Print publication date: 1899 Jan
Volume: 15
Volume Series: New Ser
Issue: 11
First page: 721
Last page: 725
License: This work is free of known copyright restrictions. For more information, please see PMC Back Issue Digitization.
License URL: https://creativecommons.org/publicdomain/mark/1.0/

==============================

ATLANTA

Medical and Surgical Journal.

Vol. XV. JANUARY, 1899. No. 11.

DUNBAR ROY, A.B., M.D., EDITOR. M. B. HUTCHINS, M.D., BUSINESS MANAGER.

### ORIGINAL COMMUNICATIONS.

(*Abstract.*)

### THE TECHNICS OF THE OPERATIVE TREATMENT OF INTESTINAL OBSTRUCTION.

Dr. Frederick Home Wiggin, of New York, read a paper with this title, it being a contribution to the discussion of the general subject of "Intestinal Obstruction." He said that the various conditions for which operations are usually demanded are: (1) Strangulation of the gut by bands, extensive adhesions or apertures; (2) volvulus; (3) intussusception; (4) obstructions due to neoplasms; (5) compression by tumors external to the gut; (6) obstruction from foreign bodies, such as gallstones and enteroliths; and (7) obstruction caused by fecal masses.

Where the case is one of acute intestinal obstruction there is but little time for preparation. The loose pieces of furniture should be removed from the room selected for the operation, and sheets wet with carbolic acid solution, one to twenty, or bichloride solution, one to five hundred, should be placed over the carpet. In this preparation of the room it is important that no dust be raised. The instruments are boiled for ten minutes in a two per cent. solution of carbonate of soda and then placed in trays containing sterilized water. The towels may be sterilized in a special sterilizer or by boiling. A large quantity of saline solution (one teaspoonful of common salt to the quart of water) should be on hand, and a wash-boiler, after thorough cleansing, should be filled with water which has been sterilized by boiling for an hour. This water is then rapidly cooled in pitchers surrounded by ice. Where there is great haste, it is admissible to take the water from the hot-water faucet. If there has been much vomiting, or if there is considerable abdominal distention, it will be well to follow Kussmaul's suggestion, to wash out the stomach with saline or boric acid solution. If, in addition, the patient is much prostrated, and does not respond well to the ordinary cardiac stimulants, from one to three pints of saline solution should be injected into the veins.

The patient having been anesthetized, the skin over the field ot operation is treated successively with the following: (1) Green soap; (2) hydrogen dioxide; (3) lathered and shaved; (4) water; (5) equal parts of alcohol and ether; (6) one to five hundred bichloride solution in alcohol; and (7) sterile water or saline solution. The bladder should then be emptied by catheter. When the site of obstruction cannot be definitely located, an incision, four inches long, should be made through the right rectus muscle, between the umbilicus and the pubes. If distended coils of intestine obscure the view, they should be aspirated or incised, and the wounds so made closed by suture, and the parts disinfected with hydrogen dioxide. The first effort of the operator should be to find the cecum. If it is greatly distended there is good reason to believe that the obstruction is in the colon, but if there is little or no distention, it is probable that the stoppage is in the small intestine. The rectum should, of course, be explored prior to the abdominal section. Where the obstruction is supposed to be in the colon, the hand should be passed over the entire length of the large bowel, or until the obstruction is found. Where the obstruction is suspected to be in the small intestine, the operator should look along the brim of the pelvis and in the region of the cecum for the collapsed portion of bowel, and follow this down to the obstruction. The various hernial orifices should also be examined, remembering that sometimes two forms of obstruction may coexist.

If the intestinal obstruction is caused by bands, these should be ligated on both sides near their attachment, and removed. If a diverticulum or an adherent appendix is the cause of the trouble, these portions of the bowel should be removed in the ordinary manner, and the opening in the gut closed with Lembert sutures. According to the writer's experience, when volvulus occurs in the small intestine it is not only safe, but desirable, to draw the intestine out of the abdomen, taking care to keep it hot and moist by wrapping it in gauze or soft towels wrung out in hot saline solution. Where an intussusception is the cause of the obstruction, the tumor should be encircled below its apex by the finger and thumb, and the sheath held a few inches lower down, while the apex of the tumor is pushed upward. Traction from above the tumor should not be employed. If the intussusception is irreducible, the following method, described by Maunsell, is recommended : A slit is made in the intussuscipiens and gentle traction is exerted on the intussusceptum until its neck appears outside the opening in the intussuscipiens. The base is then transfixed with two straight needles, armed with horsehair, and the intussusception is amputated one-fourth of an inch above the needle. The sutures are now passed through the invaginated bowel, caught up in the interior of the bowel, divided and tied. The invagination is then reduced, and the slit closed. Thanks to modern surgery, most neoplasms causing intestinal obstruction can be removed, and naturally such a course is preferable to colotomy. Where it is inadvisable to resect the portion of bowel containing the growth, an incision, four inches long, should be made over this portion, in the direction of the fibers of the external oblique, and the bowel drawn upward until its mesenteric attachment is on a level with the external incision. A slit is then made in the mesentery and a glass rod is passed through, and iodoform gauze wound around the ends of the rod. The rod is left in position until adhesions have formed, when the gut is opened.

When the intestinal obstruction results from the pressure of a neoplasm external to the gut, the new growth should be extirpated, but if this is not possible, a fecal fistula must be established above the point of obstruction. Gallstones or enteroliths causing obstruction should be pushed a little upward or downward, and then removed by an incision. The object of this is to avoid making the incision through the portion of the gut which is likely to have been damaged by pressure. Fecal accumulations causing obstruction are best removed by a high enema of saline solution, injected at a temperature of 100° F. by means of a fountain syringe raised three feet above the patient. The flow should be intermitted from time to time, as the patient complains of distention or colic. The enema should be retained as long as possible, for the object in giving it is to secure the softening of the mass, rather than to stimulate peristalsis. The procedure may be repeated several times, and its action assisted by the administration of small doses of calomel and soda.

When the gut is found to be gangrenous, in a case of intestinal obstruction, an end-to-end anastomosis should be effected, and for this purpose Dr. Wiggin prefers his modification of Maunsell's method. The modification consists in doing away with the invagination and the slit. The portion of intestine to be extirpated is emptied of its contents by pressure. The portion to be removed is then isolated by clamps on either side, and a V-shaped incision is made, having its apex in the mesentery. The mesenteric vessels are ligated before being cut, and the wound in the mesentery is sutured. After washing the divided ends of the bowel with hydrogen dioxide, they are united by two sutures passing through all the intestinal coats, the first suture being at the inferior or mesenteric border, and the second directly opposite, at the highest point. The third and fourth sutures are passed on either side half way between the first two. The two other sutures are passed in the same way, the needle going from within the gut and piercing all the coats, then back through the peritoneal, muscular and mucous coats to the interior of the other segment of bowel. The ends are then tied in the bowel. This process is continued until all the sutures but one or two are passed. For the latter Lembert sutures are substituted. If the sutures have been properly inserted and tied, the peritoneum will now be turned in and the stitches hidden.

The operation having been completed, and the dressings applied, the patient is placed between the folds of a warm blanket and only a little warm water allowed by mouth for the first twelve or eighteen hours. Then a few drachm doses of liquid peptonoids are given at intervals of twenty minutes, and, if well borne, peptonized milk is added. The tendency is to give too small quantities of food at too frequent intervals. The bowels are moved on the third or fourth day by small doses of calomel and soda.

In closing Dr. Wiggin laid great emphasis on the fact, that the prognosis in this class of cases depends more upon the promptness with which surgical treatment is instituted than upon any other factor.

---

### THE SOUTHERN SURGICAL AND GYNECOLOGICAL ASSOCIATION.

*Proceedings of the Eleventh Annual Meeting, Held at Memphis, Tennessee, December 6, 7, and 8, 1898.*

The Association met at the Gayoso Hotel, under the presidency of Dr. Richard Douglas, of Nashville, Tenn.

Prayer was offered by Bishop Gailor.

Dr. Alexander Erskine, of Memphis, delivered an "Address of Welcome" on behalf of the local profession, which was responded to by President Douglas.

After the disposal of routine business, the scientific session was opened by a paper by Dr. William E. Parker, of New Orleans, La., entitled:

### GUNSHOT WOUNDS.

He described the hospital conditions in Cuba and the wounds seen by him made by the modern bullet. He spoke of some experiments, made under the direction of Major and Surgeon L. A. LaGarde, of the regular army, as to the relative penetrating powers of the Mauser, Krag-Jorgensen and Springfield rifles.

Dr. F. W. McRae, of Atlanta, followed with a paper on
